# Retraction: Neutralizing Aptamers from Whole-Cell SELEX Inhibit the RET Receptor Tyrosine Kinase

**DOI:** 10.1371/journal.pbio.1002215

**Published:** 2015-07-17

**Authors:** Laura Cerchia, Frédéric Ducongé, Carine Pestourie, Jocelyne Boulay, Youssef Aissouni, Karine Gombert, Bertrand Tavitian, Vittorio de Franciscis, Domenico Libri

The authors and editors retract this publication following an investigation into concerns around the data presented in Figures 3 and 4 that were brought to the editors’ attention. The text below has been agreed to by the editors and authors.

Some of the lanes presented in Figure 3 (five out of six lanes of the right "Ret" panel in Fig 3B) came from an unrelated experiment that had already been published in Fig 4A of a Biochem J paper from 2003 by the de Franciscis lab, with the same first author (Cerchia et al. Biochemical Journal, 372, 897–903). As this previously published data is subject to copyright, this content has been removed from the published record of this paper. Additionally, several lanes have been duplicated both within Figure 3 and between Figures 3 and 4 of this paper.

Some of the original blots were provided to the editors as part of this investigation and the first author, LC, states that the errors occurred as a consequence of incorrect incorporation of representative blots in the figures and, together with the senior author responsible for oversight of Dr. Cerchia when the research was carried out (VdF), apologizes for this. Even though LC maintains that the conclusions remain valid, the detected image manipulation in the published figures undermines the confidence of the other authors (FD, CP, JB, YA, KG, BT & DL) and the overall editorial confidence in the results presented in the latter half of this study. It should be noted specifically that the co-authors from collaborating groups (FD, CP, JB, YA, KG, BT & DL) were not involved in the preparation of these figures, and there is no concern about the results that they contributed (Figures 1 & 2). The manipulations were not identified prior to publication and all three senior authors (BT, VdF & DL) approved the submitted manuscript.

An institutional inquiry has been undertaken at the CNR in Rome, where de Franciscis and Cerchia are both located; a retraction was deemed appropriate as a result of this inquiry.

Given the serious concerns about the validity of some of the data presented in this paper, the authors and editors are thus issuing a retraction. The authors deeply regret any inconvenience this publication has caused for others. All authors agree to this retraction.
